# Modelling of ecological status of Polish lakes using deep learning techniques

**DOI:** 10.1007/s11356-020-10731-1

**Published:** 2020-09-22

**Authors:** Daniel Gebler, Agnieszka Kolada, Agnieszka Pasztaleniec, Krzysztof Szoszkiewicz

**Affiliations:** 1 Department of Ecology and Environmental Protection, Poznan University of Life Sciences, Wojska Polskiego 28, 60-637 Poznan, Poland; 2 Institute of Environmental Protection—National Research Institute, Kolektorska 4, 01-692 Warsaw, Poland

**Keywords:** Artificial neural network, Biological indices, Macrophytes, Phytoplankton, Phytobenthos, Water quality

## Abstract

Since 2000, after the Water Framework Directive came into force, aquatic ecosystems’ bioassessment has acquired immense practical importance for water management. Currently, due to extensive scientific research and monitoring, we have gathered comprehensive hydrobiological databases. The amount of available data increases with each subsequent year of monitoring, and the efficient analysis of these data requires the use of proper mathematical tools. Our study challenges the comparison of the modelling potential between four indices for the ecological status assessment of lakes based on three groups of aquatic organisms, i.e. phytoplankton, phytobenthos and macrophytes. One of the deep learning techniques, artificial neural networks, has been used to predict values of four biological indices based on the limited set of the physicochemical parameters of water. All analyses were conducted separately for lakes with various stratification regimes as they function differently. The best modelling quality in terms of high values of coefficients of determination and low values of the normalised root mean square error was obtained for chlorophyll *a* followed by phytoplankton multimetric. A lower degree of fit was obtained in the networks for macrophyte index, and the poorest model quality was obtained for phytobenthos index. For all indices, modelling quality for non-stratified lakes was higher than this for stratified lakes, giving a higher percentage of variance explained by the networks and lower values of errors. Sensitivity analysis showed that among physicochemical parameters, water transparency (Secchi disk reading) exhibits the strongest relationship with the ecological status of lakes derived by phytoplankton and macrophytes. At the same time, all input variables indicated a negligible impact on phytobenthos index. In this way, different explanations of the relationship between biological and trophic variables were revealed.

## Introduction

When the Water Framework Directive (WFD; Directive 2000/60/EC n.d.) came into force in 2000, lake assessment based on aquatic organisms has acquired immense practical importance. Based on the assumption that various ecosystem components, called biological quality elements (BQEs), are comprised of ecosystem status and reflect different aspects of its condition, the significant development of biological monitoring methods took place, and the approach to assess the ecological status of aquatic ecosystems has become widely used. As prescribed in Annex V of WFD, many characteristics of BQEs (i.e. species composition and abundance) should be included in the assessment system, which, together with supporting physicochemical parameters, give the overall view of the ecological status of the water environment. Currently, after over a decade of vast scientific research and environmental monitoring, extensive hydrobiological databases have been gathered, which provide an opportunity to increase our knowledge about the functioning of aquatic ecosystems (Carvalho et al. 2019; Hering et al. 2010). Based on the large databases, the precise prediction of the characteristics of BQEs and forecast changes in aquatic biota under changing abiotic conditions is possible (e.g. Rocha et al. 2017). Considering that the ecological assessment is usually costly and requires extensive fieldwork efforts, ecological modelling may support water managers by providing classification results into not investigated waterbodies from the extrapolation based on a smaller set of data (Benedini and Tsakiris 2013).

An ecological approach to surface water assessment and management under WFD ensured a vast amount of ecological data obtained in freshwater monitoring programmes both at the national and European Union (EU) scale. However, the limitations of monitoring data, such as their extensiveness, variability, gaps and multiple sources of errors, can limit their effective use (Hering et al. 2010). The above characteristics of the data obtained in aquatic monitoring programmes allow them to be classified as big data (Durden et al. 2017; Hampton et al. 2013). The use of big data in various fields of science, including freshwater research, has become common in recent years (e.g. Dafforn et al. 2016; Farley et al. 2018; Li et al. 2015). Their potential to solve complex ecological issues will grow in the future as a result of an increase in the existing data and the broader use of new analytical methods (Hallgren et al. 2016; LaDeau et al. 2017; Secchi 2018). It must, however, be stressed that all of the disadvantages of big datasets also require the use of adequate analytical tools, such as random forests, genetic algorithms and deep learning methods, which are based on artificial neural networks (Benedini and Tsakiris 2013; Secchi 2018; Shi 2018; Sun and Scanlon 2019). The deep learning technics have the potential to be applied to diverse research of any aquatic organisms (Iqbal et al. 2019; Joutsijoki et al. 2014; Tiyasha et al. 2020) as well as water quality issues (Alizadeh et al. 2018; Kargar et al. 2020; Li et al. 2015; Zhu et al. 2019). Models based on artificial neural networks are recommended to solve complex and nonlinear relationships in ecological study (Park and Lek 2016) and often provided more efficient results compared with the classical modelling techniques (Heddam 2016; Wu et al. 2014). Both, big data and machine learning techniques, can also be effectively used in environmental and water management (Sun and Scanlon 2019).

The WFD-compliant lake monitoring in Poland has started in 2008, and initially it included phytoplankton and macrophytes as the only biological elements. These methods included the Phytoplankton Multimetric for Polish Lakes (PMPL; presented in Hutorowicz and Pasztaleniec 2014) and the Ecological State Macrophyte Index (ESMI; presented in Ciecierska and Kolada 2014). The method based on benthic diatoms, the Diatom Index for Lakes (IOJ; Picinska-Fałtynowicz and Błachuta 2010), has been introduced in routine lake monitoring since 2010 (Kolada et al. 2016). The primary producers are strongly influenced by eutrophication and are known to respond to changes in both abiotic and biotic conditions clearly (Lyche-Solheim et al. 2013). In the assessment of the ecological status, these elements are complementary. The response of elements with a short-generation time, i.e. phytoplankton and benthic diatoms, to water nutrient enrichment is rapid and direct, but it could be temporary. In contrast, macrophytes respond slowly but they mirror long-term trends. Within the monitoring of these elements, data from several hundred lakes have already been collected so far, and the amount of data is growing with each subsequent year. The other biological indices required for the WFD-compliant monitoring, i.e. these based on macrozoobenthos and fish, have been elaborated relatively recently in Poland and are available from a limited number of lakes so far. They are also not sufficiently verified and tested on a national level; therefore, they were not analysed in this study.

The study aimed to challenge modelling of the ecological status of lakes based on the five fundamental eutrophication parameters of water using artificial neural networks (ANNs). We attempted to reveal the major environmental variables predicting the pattern of autotroph communities. Additionally, we explored whether the data gathered in national monitoring programmes can be efficiently used in ecological modelling, providing good quality predictive models and showing possible application for water management. We hypothesised that the deep learning techniques based on selected, easily measurable, physicochemical parameters of water could efficiently and accurately estimate the values of ecological status indices, which could not be reached by a traditional statistical approach. Moreover, we analysed the impact of eutrophication on various groups of aquatic autotrophs regarding the stratification regime (type of water mixing)—the main feature of the lake abiotic typology (Kolada et al. 2005, 2017). We hypothesised that neural networks and traditional statistical approaches deliver different information on biological reaction to habitat factors in the lake ecosystem. Moreover, we expected a distinctive reaction of various groups of aquatic autotrophs to habitat variables.

## Materials and methods

### Data collection

**Fig. 1:**
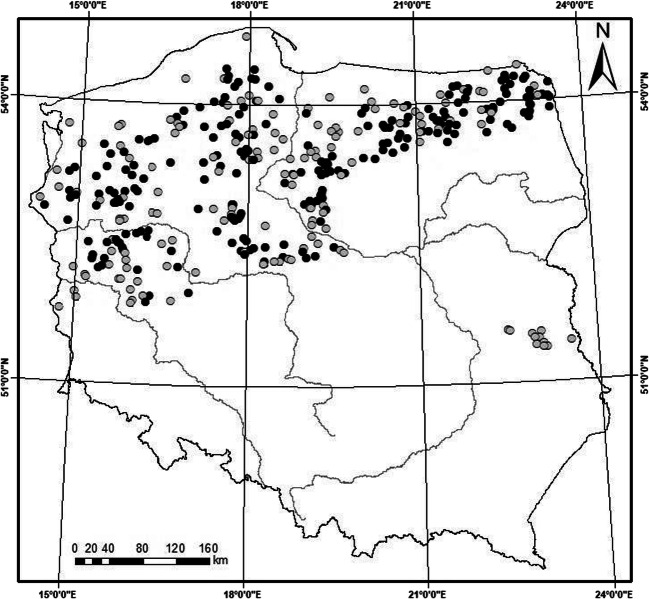
Location of the study sites within the Polish lakelands; black circles, stratified lakes (*n* = 221), and grey circles, non-stratified lakes (*n* = 145)

Our analyses were based on 393 records (lake-years) collected from 366 lakes located within the Polish lake districts (Fig. 1). The selection and number of lakes used in this study provide full representation of abiotic conditions of lakes monitored under WFD and cover the entire range of their geographical distribution in Poland. All of the analysed lakes are lowland (≤ 200 m a.s.l.), with non-coloured highly alkaline waters (> 1.0 meq L^-1^), but they differ in trophic conditions (Appendix Tables 3 and 4). Of them, 221 lakes (60%) stably stratify in the summer period (stratified lakes), while 145 are permanently mixed (polymictic lakes). The national monitoring data on water physicochemical parameters and three main groups of plant organisms, i.e. phytoplankton, phytobenthos and macrophytes, collected in the years 2010–2015 were used in the study. The survey period covers the second River Basin Management Plan and the bioassessment results were fully verified and reported to European Environmental Agency. Our study included four biological indices: chlorophyll *a* concentration, the phytoplankton index PMPL, the macrophyte index ESMI and the phytobenthos index IOJ. We focused on primary producers, i.e. phytoplankton, phytobenthos and macrophytes, because their monitoring has been carried out the longest among all the BQEs (Kolada et al. 2016) and the availability of data is sufficient for the use of artificial neural networks (ANNs) in ecological status modelling (e.g. Gebler et al. 2017). The phytoplankton, phytobenthos and macrophyte indices have been sufficiently verified and tested on a national level (Kolada et al. 2016) as well as internationally intercalibrated in the pan-European intercalibration exercise (European Commission 2011; Kelly et al. 2014; Phillips et al. 2014; Portielje et al. 2014).

Since lakes with various stratification regimes function differently, the analyses were performed for records from stratified (*n* = 237) and non-stratified lakes (*n* = 156), separately. The significant vertical variation of water temperature, observed particularly in the pelagic zone of deep lakes, is a characteristic feature of lakes in the temperate zone. A thermal stratification is one of the most important factors affecting chemical and physical processes, decisive for nutrient availability, thus regulating ecological functioning, i.e. phytoplankton community abundance, structure and composition during the summer (Yang et al. 2016). The essential role of stratification in the functioning of the ecosystem makes it one of the main criteria of the lake abiotic typology, also in Poland (Kolada et al. 2005, 2017).

Lakes were sampled for physicochemistry and phytoplankton at least four times during the vegetation season, from March to October (spring mixing, early summer, the peak of the summer stagnation and autumn mixing). The physicochemical parameters of the water were sampled and analysed using standard protocols applied in routine lake monitoring in Poland. The list of physicochemical parameters we used was limited to fundamental eutrophication variables. They consist of total phosphorus (TP), total nitrogen (TN), Secchi disk reading (SD), conductivity (Cond.) and oxygen concentration (O_2_): the mean hypolimnion saturation with oxygen at the peak of summer stagnation (for stratified lakes; Appendix Table 3) or oxygen content at the bottom in the summer (for non-stratified lakes; Appendix Table 4). In the study we used seasonal mean of all of these parameters.

For the quantitative analysis of phytoplankton, water samples were taken in the deepest part of a lake according to a harmonised national protocol (Hutorowicz 2009). In stratified lakes, during the summer stagnation period, integrated water samples were collected from the epilimnion layer, and in the spring and autumn, from the euphotic layer. In polymictic lakes, integrated samples were taken from the layer between 0 and 5 m. The quantitative analyses of phytoplankton followed the standard Utermöhl method (1958). The phytoplankton multimetric PMPL is composed of three metrics: “Chlorophyll *a*”, “Total Biomass” and “Biomass of Cyanobacteria” (for details see Hutorowicz and Pasztaleniec 2014).

In addition to the multimetric PMPL, we used its component chlorophyll *a* (Chl*a*) separately, as this measure is one of the most commonly used parameters in lake assessment practice worldwide (Pasztaleniec 2016). The chlorophyll *a* concentration was analysed according to the spectrophotometric method (PN-86/C-05560/02).

Macrophytes were investigated once a year, at the peak of the vegetation season (from mid-June to mid-September) using the belt transect method (Kolada et al. 2014). The macrophyte multimetric ESMI is composed of three main components: the Pielou’s index of evenness (Pielou 1975) and the colonisation index Z, which is a ratio of a total vegetated area and a lake area with a depth of less than 2.5 m (for details see Ciecierska and Kolada 2014).

All lakes were studied for benthic diatoms once a year using a standardised procedure (Kelly et al. 2014; Picinska-Fałtynowicz and Błachuta 2010). For the majority of the lakes, samples were taken in late summer/autumn and for nearly 20% of lakes, in spring/early summer. The phytobenthos multimetric IOJ is calculated as a weighted mean of two modules: a trophic index derived from the diatom trophic values according to Schaumburg et al. (2007), weighted by the factor 0.6 and the module of reference species weighted by the factor 0.4 (for details see Kelly et al. 2014).

### Artificial neural networks

**Fig. 2:**
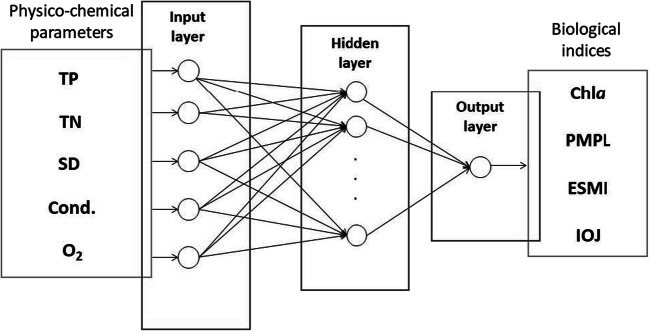
Artificial neural networks structure for the addressed problem

In the modelling of four biological indices, a deep learning technique based on the artificial neural network was used. In our investigation, we used the multi-layer perceptron (MLP) type of network, which is commonly used in water quality modelling (Tiyasha et al. 2020). The MLP has many advantages such as self-adaptive iterative algorithms, highly flexible function approximator, no need to know the mathematical structure of the relationships studied and prior knowledge of them, and the possibility of using in both linear and nonlinear relationships. Of the various types of methods, the MLP is often dedicated to nonlinear and complex data usually faced in ecological studies (Park and Lek 2016). In our study, the three-layer MLP was used (Fig. 2). The input layer included five neurons corresponding to five water quality parameters (TP, TN, SD, Cond., O_2_). In the output layer, there was one neuron corresponding to each modelled biological index (Chl*a*, PMPL, ESMI and IOJ). The number of neurons in the hidden layer was determined in the learning process; according to recommendations by Fletcher and Goss (1993), it ranged from 5 (2*n*^1/2^ + *m*) to 11 (2*n* + *m*), where *n* is the number of input neurons and *m* the number of output neurons. The algorithm of Broyden-Fletcher-Goldfarb-Shanno (BFGS) was used to adjust the weights of the networks. Among other algorithms that are available in the STATISTICA software (Scaled Conjugate Gradient and Gradient Descent), the BFGS algorithm allowed to obtain the best quality models (Dell Inc. 2016). Before the ANN learning, the database was divided into three independent subsets. The training dataset used in the first phase of ANN learning consisted of 70% of the data. For the validation and testing dataset, 30% of research records were used (15% in each dataset). The testing dataset was used only for final model evaluation, and it was not available for the learning process.

Prior to the modelling, the r-Pearson correlation coefficient was also calculated to determine whether the input variables were correlated with each other (Appendix Tables 5 and 6; Dormann et al. 2013). The correlation was also calculated to test the relationships between physicochemical parameters and biological metrics. Due to the different ranges of the variables used, all input and output variables were standardised. This allows to avoid the problem of misinterpretation of the impact of variables resulting not from existing relationships but as an effect of high variation and different units (Park and Lek 2016). For the input variables, the autoscaling was used (Eq. 1) as recommended for environmental variables in ecological studies. For outputs, the min-max normalisation within 0.1–0.9 range was used (Eq. 2).

1 $$ {z}_i=\frac{x_i-\mu }{\sigma } $$

where *x*_*i*_ is the i-th value of each input variable, *z*_*i*_ is the i-th standardised value of the variable, *μ* is the mean of the variable and *σ* is the standard deviation of the variable.

2 $$ {y}_i^{\prime }=\frac{\left({y}_{\mathrm{i}}-{y}_{\mathrm{min}}\right)}{y_{\mathrm{max}}-{y}_{\mathrm{min}}}\left({y}_{\mathrm{min}}^{\prime }-{y}_{\mathrm{max}}^{\prime}\right)+{y}_{\mathrm{min}}^{\prime } $$

where *y*_*i*_ is the i-th value of output variable; ${y}_i^{\prime }$ is the i-th normalised value of variable; *y*_*min*_ and *y*_*max*_ are the minimum and maximum values of output variable, respectively; and ${y}_{min}^{\prime }$ and ${y}_{max}^{\prime }$ are the minimum and maximum normalised values of output variable, respectively.

The performance of each network was evaluated using the coefficient of determination (*R*^*2*^; Eq. 5), which describes the proportion of the variance of output data explained by the model. Additionally, the root mean square error (*RMSE*; Eq.4) and the normalised root mean square error (*NRMSE*; Eq. 5) were calculated on the basis of values of biological indices modelled by the networks and calculated on the basis of the botanical research. These three evaluation criteria are among the most common used in quantification of model quality (e.g. see overview presented by Moriasi et al. 2007). Therefore, they are considered as easy to interpret results and enable wide comparison with other studies.

3 $$ {R}^2=1-\frac{\sum \limits_{i=1}^n{\left({y}_i^{\prime }-{\hat{y}}_i^{,}\right)}^2}{\sum \limits_{i=1}^n{\left({y}_i^{\prime }-{\overline{y}}_i^{\prime}\right)}^2} $$

4 $$ RMSE=\sqrt{\frac{\sum \limits_{i=1}^n{\left({y}_i^{\prime }-{\hat{y}}_i^{,}\right)}^2}{n}} $$

5 $$ NRMSE=\frac{RMSE}{y_{\mathrm{max}}^{\prime }-{y}_{\mathrm{min}}^{\prime }} $$

where ${\hat{y}}_i$ is the i-th normalised value of output variable derived from the models, ${\overline{y}}_i^{\prime }$ is the mean of the empirical value of each output variable, and *n* is the number of repititions.

To determine the effect of each input variable on the dependent output variables, sensitivity analysis was applied. It is one of the analytical methods, which shows the importance of predictive variables for a model (Park and Lek 2016). The value obtained for each variable is the ratio of the mean square error of the network without this variable and the error with a set of all explanatory variables.

## Results

### Relationship between biological metrics and physicochemical parameters

The r-Pearson correlation coefficients between environmental input data and biological metrics were the highest between SD and phytoplankton index PMPL in both non-stratified (−0.79) and stratified lakes (−0.77) (Table 1). A significant and relatively strong correlation was also detected between SD and macrophyte index ESMI as well as between SD and chlorophyll *a*. Significant correlations were also found between most of the considered biological indices and nutrients (TN and TP) as well as conductivity, but these relationships were weaker. The lowest coefficient values and, thus, the weakest links between variables were obtained for the phytobenthos index IOJ.

**Table 1:** r-Pearson correlation coefficient between biological indices and physicochemical parameters (*p < 0.001, **p < 0.01, ***p < 0.05)

Biological indices		Lake mixing type	Physicochemical parameters				
			TP	TN	SD	Cond.	O_2_
Chlorophyll *a*	Chl*a*	Stratified	0.58**	0.59*	−0.69*	0.52*	−0.15***
		Non-stratified	0.59*	0.69*	−0.68*	0.44*	0.05
Phytoplankton multimetric	PMPL	Stratified	0.45*	0.51*	−0.77*	0.42*	−0.22*
		Non-stratified	0.41*	0.63*	−0.79*	0.36*	0.05
Macrophyte multimetric	ESMI	Stratified	−0.40*	−0.46*	0.65*	−0.38*	0.05
		Non-stratified	−0.34*	−0.46*	0.71*	−0.26**	−0.02
Phytobenthos index	IOJ	Stratified	−0.38*	−0.19**	0.15***	−0.29*	0.03
		Non-stratified	−0.42*	−0.05	0.22**	−0.29*	0.13

Values of r-Pearson correlation coefficient showed no collinearity (r < 0.70) between the five water quality parameters (Appendix Tables 5 and 6). Correlations between five input variables were low both for stratified and non-stratified lakes. The level of correlation detected did not indicate a disturbance of the neural network analyses; thus, they were all used further as predictors.

### Performance of models

**Fig. 3:**
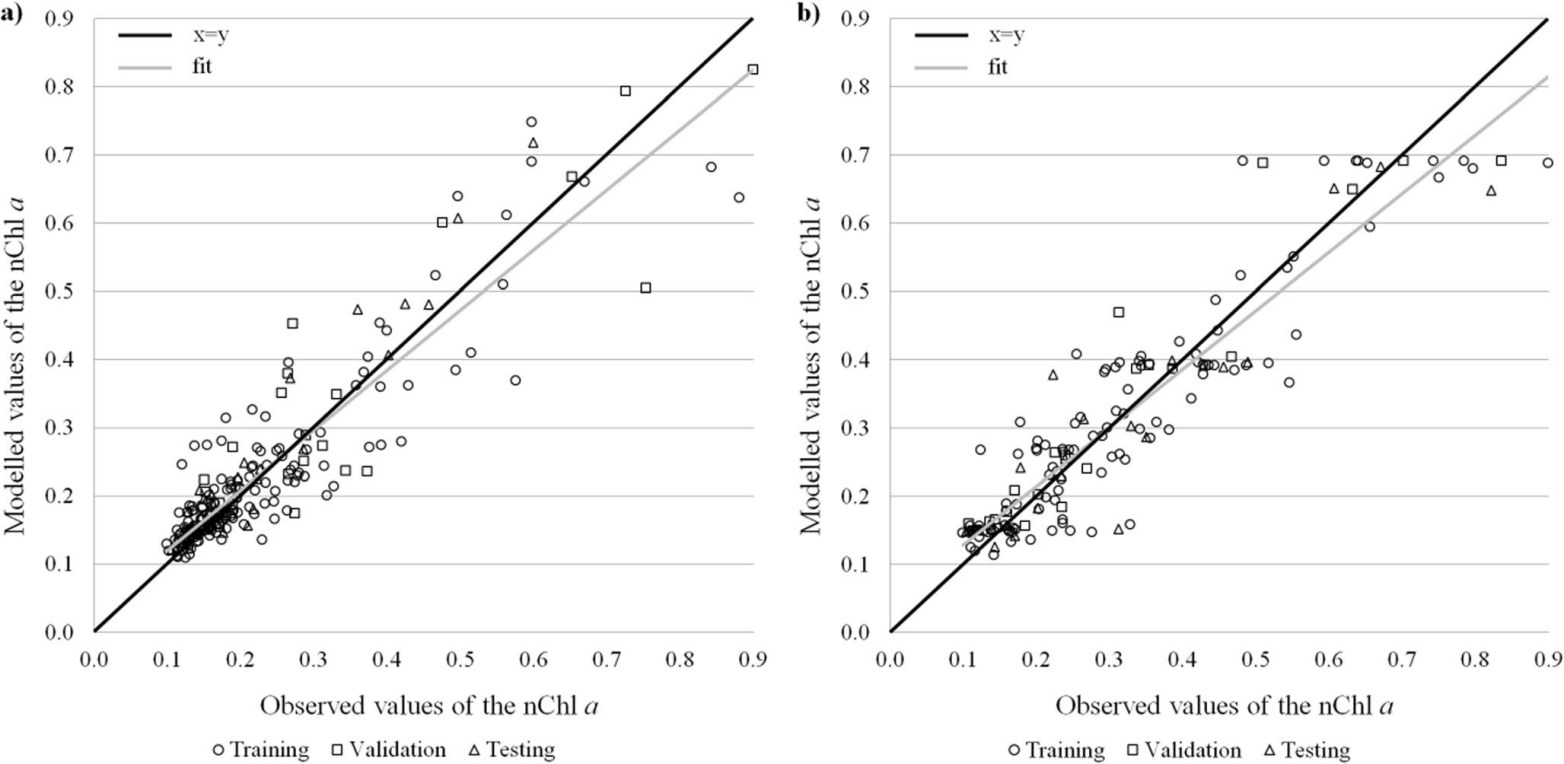
Modelled and observed normalised values of the chlorophyll *a* concentration in stratified (**a**) and non-stratified (**b**) lakes

**Fig. 4:**
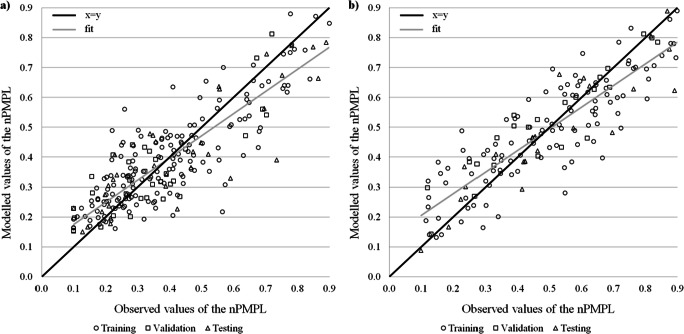
Modelled and observed normalised values of the phytoplankton index—PMPL in stratified (**a**) and non-stratified (**b**) lakes

**Fig. 5:**
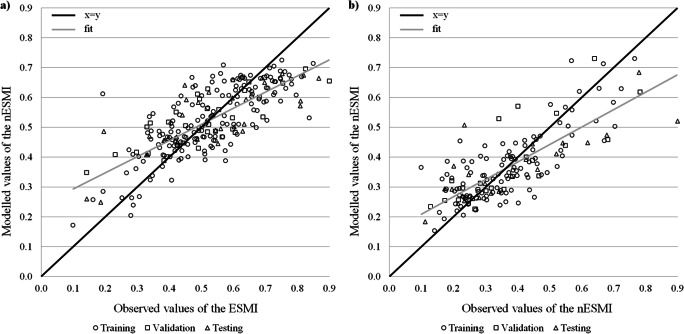
Modelled and observed normalised values of the macrophyte index—ESMI in stratified (**a**) and non-stratified (**b**) lakes

**Fig. 6:**
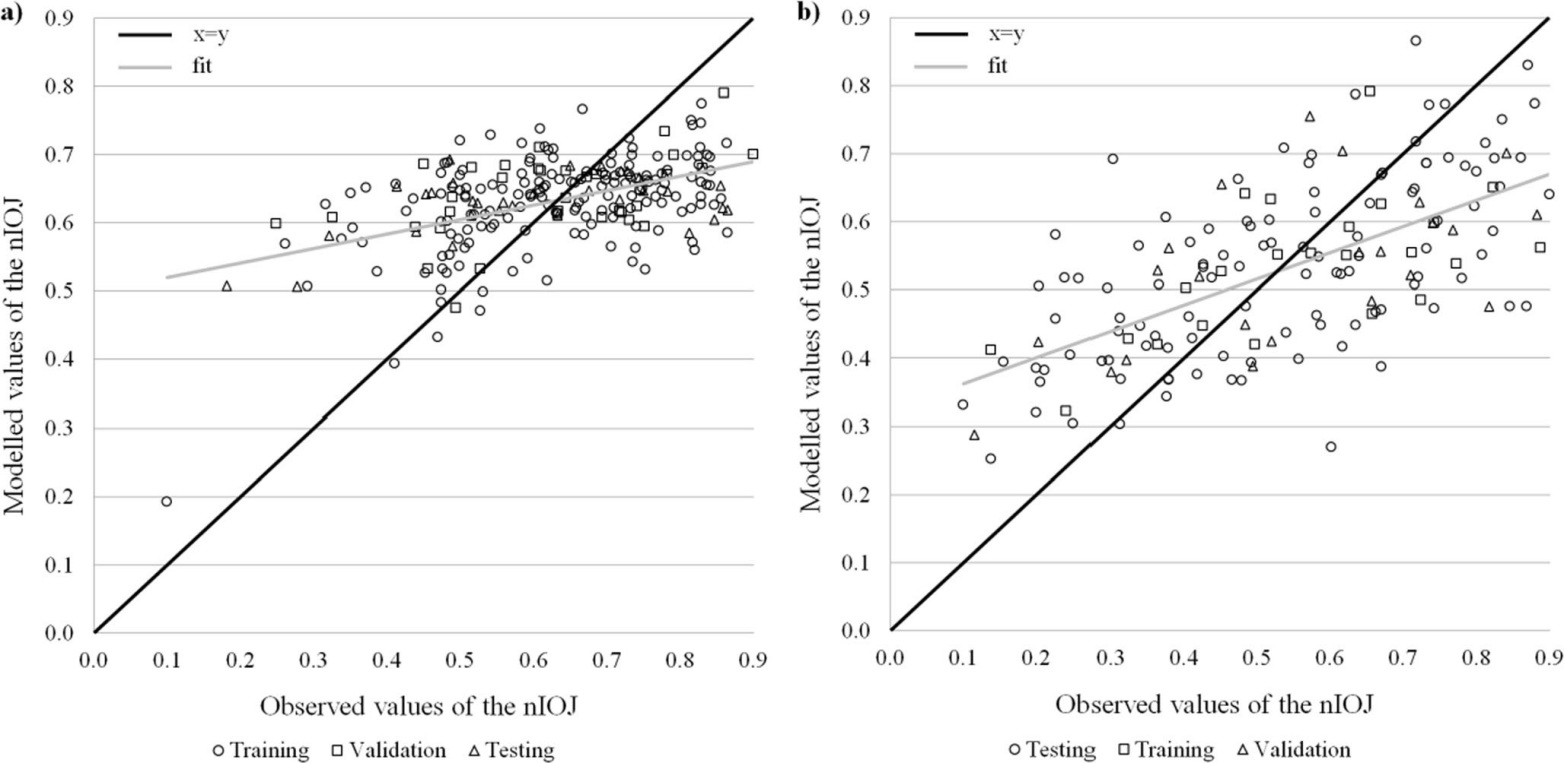
Modelled and observed normalised values of the phytobenthos index—IOJ in stratified (**a**) and non-stratified (**b**) lakes

For each modelled index and each lake type, one network with the lowest mean square error and the highest coefficient of determination, which gives the fraction of explained variance of the analysed dataset, was chosen. The quality of the eight models for the three processes of network learning has been shown in Table 2 and graphically summarised in Figs. 3–6.

**Table 2:** Performance parameters of the artificial neural network models for computation of four ecological status indices (*number of neurons in three layers: input → hidden → output)

Index	Dataset	ANN-structure*	*R*^*2*^	RMSE (NRMSE)	ANN-structure*	*R*^*2*^	RMSE (NRMSE)
		Stratified lakes			Non-stratified lakes		
Chl*a*	Training	5→7→1	0.824	0.056 (7.1%)	5→8→1	0.853	0.067 (8.4%)
	Validation		0.843	0.077 (9.8%)		0.887	0.066 (9.1%)
	Testing		0.851	0.046 (9.4%)		0.861	0.071 (9.8%)
PMPL	Training	5→7→1	0.722	0.100 (12.6%)	5→9→1	0.809	0.092 (11.7%)
	Validation		0.764	0.100 (12.9%)		0.829	0.085 (11.9%)
	Testing		0.737	0.119 (15.1%)		0.807	0.100 (12.7%)
ESMI	Training	5→6→1	0.589	0.096 (12.8%)	5→10→1	0.610	0.089 (13.4%)
	Validation		0.562	0.106 (14.0%)		0.638	0.100 (15.4%)
	Testing		0.570	0.117 (16.2%)		0.571	0.133 (16.9%)
IOJ	Training	5→6→1	0.243	0.126 (16.5%)	5→10→1	0.395	0.159 (19.9%)
	Validation		0.220	0.131 (20.1%)		0.344	0.150 (20.0%)
	Testing		0.193	0.156 (22.8%)		0.357	0.165 (21.4%)

Among the four biological indices, networks for Chl*a*, both in stratified and non-stratified lakes, had the highest precision (Table 2). For these two networks, the values of the *R*^*2*^ exceeded 0.8 at every phase of the network learning. For the final testing, which was based on independent calibration data, the determination ratio in the two lake types was 0.851 and 0.861, respectively. This means that over 85% of the variance of the modelled variable has been explained by these two models. Furthermore, the NRMSE values for these models were lower than 10%. The relation between observed and modelled values of Chl*a* was strong (Fig. 3a and 3b). It can also be noted that the fitness of the models is close to the expected regression line. The quality of models for phytoplankton multimetric PMPL was lower compared with its single component Chl*a*. The performance parameters for stratified and non-stratified lakes achieved by the models were 0.737 and 0.807, respectively, in the testing stage of network learning process. Nevertheless, these networks were the last, with the explained variance above 70 and 80%, and relatively good fitness of modelled values was observed (Fig. 4a and 4b). The normalised errors of both PMPL modelling networks exceeded 10%.

The macrophyte index ESMI performed weaker compared with networks for both phytoplankton indices, PMPL and Chl*a*. The coefficient of determination in the testing phase was about 0.570 for both types of lakes, explaining less than 60% of the variability. Moreover, the normalised errors were around 15% for these networks. The fitness of the modelled values is shown in Fig. 5a and 5b.

For the phytobenthos index IOJ, the model quality was significantly lower than for the other three biological indices, and the relation between observed and modelled values of the IOJ was relatively weak (Fig. 6a and Fig. 6b). In both analysed networks, models explained less than 20% (stratified lakes) and less than 36% (non-stratified lakes) of variance in the testing procedure. Moreover, NRMSE in both cases exceeded 20%.

It can also be noted that modelling quality for non-stratified lakes was higher than this for stratified lakes, giving a higher percentage of variance explained by the networks and lower values of errors. The most significant differences concerned the IOJ index, for which variance explained for non-stratified lakes varied between 12.4 and 16.4%, depending on the stage of learning of the network. For the other indices, the differences were lower than 10% of the explained variance, and they were the weakest for Chl*a* not exceeding 5% (1.0–4.4%).

### Sensitivity analysis

**Fig. 7:**
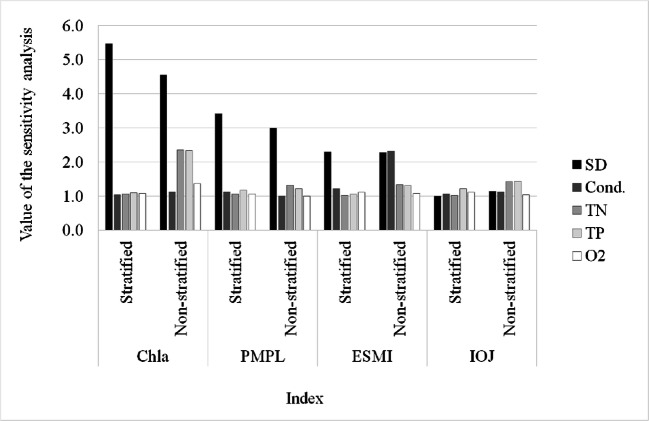
Sensitivity analysis for all constructed ANNs based on five biological indices (Chl*a*, PMPL, ESMI and IOJ) and five physicochemical variables in two mixing types of lakes

Sensitivity analysis demonstrated that the prediction models for Chl*a* and PMPL and, to some extent, for ESMI, were primarily sensitive to changes in water transparency (Fig. 7). These results correspond well with the quality of the networks. Networks, for which Secchi disk values were the most essential predictors, achieved the best prediction quality. For the phytoplankton indices, the removal of this variable from the models would cause an increase in error from three to more than five times. For the majority of the other predictors, the values were around 1, indicating their low impact on the models. The exception was the second network for Chl*a*, where the strong effect of total phosphorus and total nitrogen, next to the Secchi disk values, was also observed. The apparent dominance of Secchi disk measures as a predictor may also result from numerous relationships between most of the analysed physicochemical parameters (Appendix Tables 5 and 6). Although these parameters were not collinear, they were related to each other and represented the same signal (information) to the network. The information was represented primarily by water transparency and was not doubled by other parameters.

Conductivity seems to be a more reliable predictor in the modelling of ESMI in non-stratified lakes compared with stratified ones. In the sensitivity analysis, higher values were obtained for conductivity than for Secchi disk, whereas SD remained the most crucial predictor in stratified lakes. For the IOJ network, meanwhile, low values obtained in the sensitivity analysis for all input variables indicate the negligible impact of these predictors on the models, which also corresponded to the low quality of these neural networks. In all of the analysed networks for each index, oxygen weakly contributed to the models.

## Discussion

Our study showed that national monitoring programmes carried out under WFD requirements can be significant sources of freshwater research data. Despite various disadvantages of data gathered in broad monitoring programmes (Hering et al. 2010), they can provide valuable information about the state and functioning of aquatic ecosystems (e.g. Carvalho et al. 2019; Gebler et al. 2017; Kelly et al. 2016; Kolada et al. 2016). On the contrary, it is argued in various fields that a smaller number of data, which is more comprehensive and accurate, may be a more useful research material (Faraway and Augustin 2018; O’Hare et al. 2020; Whitaker 2018). The use of monitoring data for Polish lakes has enabled the creation of models for four indicators of ecological status. It was stressed that the use of appropriate analysis methods can also significantly increase the possibilities of using this type of data (Secchi 2018; Shi 2018). The use of one of the recommended methods, deep learning techniques (Sun and Scanlon 2019), provided efficient results giving valuable information for environmental and water management. The simple relationships demonstrated in many studies (Hutorowicz and Pasztaleniec 2014; Kolada et al. 2016) as well as in our research based on the correlation analysis (Table 1) can be significantly supplemented by the results obtained using artificial neural network that brings complementary information as emphasised also by Park and Lek (2016).

The r-Pearson correlation between biological indicators and environmental variables showed a particularly strong relationship between water transparency (SD) and phytoplankton indices (PMPL, Chl*a*). Transparency was also strongly associated with the macrophyte index (ESMI). Nevertheless, all these indicators were also related to nutrients and conductivity. The effect of nitrogen was more substantial than that of phosphorus. Extensive studies carried out by Kolada et al. (2016) based on analyses of 256 lakes surveyed in 2010–2013 showed similar trends demonstrating a relevant correlation range between biological indicators and nutrients to that of our research.

Ecological status assessment indices based on three main groups of aquatic plants showed the various capability to be modelled on the basis of physicochemical parameters of waters in the following order: phytoplankton > macrophytes > phytobenthos. The highest model precision was obtained for both phytoplankton indices, including the best quality for Chl*a*, which is a parameter that has been widely used in lake monitoring and classification schemes as a quick and easy-to-measure indicator of trophy (e.g. Carlson 1977). It is currently the most common element of ecological status assessment methods (Carvalho et al. 2013; Pasztaleniec 2016). In contrast to Chl*a*, the PMPL is a multimetric index consisting of three components (“Chlorophyll *a*”, “Total Biomass” and “Biomass of Cyanobacteria”), which represent a different approach to the ecological degradation assessment. As PMPL provides more complex information about the phytoplankton community (including both abundance and taxonomic composition) than chlorophyll *a* alone, it exhibits not only a strict relationship with trophy parameters but also reflects the level of lake ecological degradation (Hutorowicz and Pasztaleniec 2014). As pointed out by Reynolds (2000), there is no single variable or relationship that will predict the taxonomic composition of the phytoplankton. It is extremely difficult to separate the influence of water chemistry compounds on specific taxa within complex, environmental matrix, which includes also physical water mixing, light availability, carbon dynamics and biotic interactions. For this reason, input variables that mainly represent trophic degradation were not sufficient for better quality modelling of PMPL, as well as of ESMI and IOJ.

The quality of the networks for both phytoplankton indices, however, was comparable with similar ANN models for phytoplankton (e.g. Shamshirband et al. 2019; Tian et al. 2019; Wu et al. 2014). Compared with other studies on the modelling of ecological status indices based on macrophytes in rivers (Gebler et al. 2017, 2018), our models for macrophyte index in lakes had a similar quality. Model quality for both phytoplankton indices can be taken as efficient or very good and for macrophyte index as satisfactory or good (Moriasi et al. 2007). Moreover, a higher quality of all networks was obtained for non-stratified lakes, and the most significant differences of model performance were for PMPL modelling. Better relationships between this index and water quality in non-stratified lakes were also indicated by correlation analysis (Hutorowicz and Pasztaleniec 2014).

The water mixing pattern highly influences the dynamics of algae population development by determining a complex group of drivers (i.e. depth of euphotic layer and the prevalence of N and P limitations) (Reynolds 2000). Generally, the phytoplankton abundance response to nutrients increases significantly as depth decreases, and deep lakes are less responsive to nutrient enrichment (Phillips et al. 2008). On the other hand, for very shallow lakes, interactions with macrophytes are also likely to be important and top-down control mediated through zooplankton grazing is likely to play a key role in reducing planktic algae in lakes dominated by macrophytes. Kufel (1999), based on a Great Masurian Lakes study, showed a strong correlation between chlorophyll *a* and total phosphorus or SD in deep, stratified lakes, whereas such a relationships were not found in shallow macrophyte-dominated lakes. Moreover, the strength of the relationship between nutrient concentrations and phytoplankton abundance (expressed as chlorophyll *a* as well as phytoplankton biomass) depends on a range of variables and the relationship is linear; in lower nutrient concentrations at higher ranges, the relation appears to be asymptotic (Borics et al. 2013; Phillips et al. 2008). The ANN seems to be a powerful technique for modelling such complex relationships, especially in situations when the relationship is non-linear (Chen and Billings 1992).

The development of macrophytes was determined strongly by water transparency according to ANN, although it was slightly less evident than for phytoplankton. This was particularly evident in shallow waters, where SD revealed to be a comparable predictor as conductivity basing on sensitivity analysis. The relationship between conductivity and macrophyte development is generally strong since this factor reflects the trophic state of lakes well (Stefanidis and Papastergiadou 2019; Szoszkiewicz et al. 2014; Toivonen and Huttunen 1995). Generally, the water transparency does have a large influence on submerged macrophytes, whereas emergent plants are less strongly influenced by underwater conditions (Middelboe and Markager 1997; Stefanidis and Papastergiadou 2019; Toivonen and Huttunen 1995). Therefore, shallow lakes are generally more abundant in emergent plants, which are less dependent on water transparency than submerged ones, and the impact of other trophy-related metrics is more evident.

The response of benthic diatoms to environmental factors was weak based on both r-Pearson correlation and ANN. Generally, diatoms are regarded as good indicators of ecological status reacting to nutrients, dissolved inorganic carbon, conductivity and calcium (Cellamare et al. 2012; Kelly et al. 2008). Although habitat conditions are not always quickly reflected by macrophytes and benthic algae, diatoms with a short-generation time usually closely follow environmental parameters. Thus, diatoms reflect temporary water chemistry changes, whereas macrophytes follow long-term ecological tends (Cellamare et al. 2012; Schneider et al. 2012). In our study benthic diatoms did not reflect water transparency, which was obviously the most apparent habitat pattern revealed by planktonic algae and macrophytes. Moreover, the ANN was not able to identify environmental drivers influencing diatom communities. One of the reasons for the poor performance of the model for IOJ may be the Polish sampling protocol, which requires phytobenthos sampling from stable substrata (preferably emerged macrophytes or stones) from the depth of at least 30 cm below water level (Picinska-Fałtynowicz and Błachuta 2010). In fact, phytobenthos is sampled from the depth of exactly 30 cm, which is probably insufficient to capture the effect of water visibility on the phytobenthic community. It should be emphasised that in our dataset the Secchi disk visibility hardly reached the depth of less than 30 cm irrespective of the lake ecological status (Appendix Tables 3 and 4) and in lakes in bad or poor status, the SD was at least 30 cm providing favourable light conditions for diatom development at this depth. Other reasons for the lower quality of networks for the IOJ can be a limited set of explanatory variables and the lack of some habitat parameters, e.g. calcium content, which may be important for the development of these organisms as demonstrated in other studies (Fidlerová and Hlúbiková 2016; Kolada et al. 2017; Mao et al. 2018).

The use of deep learning techniques as artificial neural networks revealed a different pattern in biological response to habitat factors in the lake ecosystem than that obtained with the use of the traditional statistical approach. Sensitivity analysis strongly exposed water transparency expressed by Secchi disk depth as the principal incentive responsible for the differentiation of phytoplankton and macrophyte indices. The information based on r-Pearson correlation showed that Chl*a*, PMPL and ESMI are significantly correlated with transparency and also with nutrients and conductivity. The correlation was generally the strongest with SD, but (1) the level of correlation, even though significant, was not very convincing, and (2) the level of correlation with nutrients was also very high, especially with total nitrogen (which was even more influential in the case of Chl*a* in shallow lakes). The statistical pressure-response relationship between phosphorus and phytoplankton biomass (Chl*a*) is often stronger than that for nitrogen; however, in regions where lakes with low N/P ratio predominate, nitrogen is often a better predictor of phytoplankton biomass, particularly in non-stratified lakes (Dolman et al. 2016).

The methods used in our study managed to avoid confusion in the interpretation of correlation analysis resulting from the synergistic effect of algal growth on water transparency (Kolada et al. 2016) as well as the synergistic impact of nutrients and conductivity (which can be used as a general measure of the trophic state of lakes; Toivonen and Huttunen 1995) on water transparency. Ultimately, the use of ANN allowed connections and synergies to be resolved and reveal that water transparency is a principal direct element of the habitat, which determines biota development. Moreover, another study showed that Secchi disk transparency can be also predicted efficiently based on chlorophyll *a* concentration (Heddam 2016), and the use of Secchi disks was also reported as an efficient method for estimating the depth of a euphotic zone (Luhtala and Tolvanen 2013).

## Conclusions

The relationship between biological and environmental variables was explained differently under deep learning modelling and traditional statistical approaches. The use of the neural network technique revealed that the phytoplankton and macrophyte patterns exceptionally depend on physical factors (water transparency), whereas r-Pearson analysis indicated the comparable influence of various factors such as transparency, nutrients and conductivity.

A strong impact of water transparency on phytoplankton and to some extent on macrophytes is particularly clear in deep lakes. In shallow lakes, where light can effectively penetrate the entire water column, the water transparency gradient is less evident, and its impact on macrophyte growth is less influential. In contrast, a strong reaction of conductivity was revealed. The relationships between habitat variables collected during yearly monitoring and summer-collected benthic diatoms appeared weak or absent.

In our study, despite a limited number of input variables that characterise trophic degradation, we obtained a good quality of models for the three of four biological indices. We can expect, however, that employing of other environmental variables could improve the quality of models, especially in the case of diatom index. Other variables, e.g. calcium content, can influence plant development and biological indices calculated on their basis. Further studies should include larger scope of ecological variables, which may deliver more comprehensive picture of relationships existing in lake ecosystems.

## Data Availability

The datasets used and analysed during the current study are available from the corresponding author on reasonable request.

## Appendix

**Table 3:** Basic statistics of physicochemical parameters and biological indices for stratified lakes (*n* = 237)

Parameter	Abbreviation	Unit	Min	Max	Mean	SD	CV
Conductivity	Cond.	μS·cm^-1^	143	723	349	121	0.35
Hypolimnion oxygenation	O_2_	%	0.00	90.60	7.93	14.68	1.85
Secchi disk—water transparency	SD	m	0.50	6.40	2.73	1.30	0.47
Total nitrogen	TN	mg N·dm^-3^	0.42	5.35	1.25	0.63	0.50
Total phosphorus	TP	mg P·dm^-3^	0.003	0.45	0.05	0.05	0.99
Multimetric Diatom Index for Lakes	IOJ	-	0.29	0.94	0.72	0.12	0.17
Ecological State Macrophyte Index	ESMI	-	0.09	0.91	0.52	0.16	0.31
Phytoplankton Multimetric for Polish Lakes	PMPL	-	0.00	4.86	1.81	1.22	0.67
Chlorophyll *a*	Chl*a*	μg/l	0.8	102.0	17.1	18.1	0.94

**Table 4:** Basic statistics of physicochemical parameters and biological indices for non-stratified lakes (*n* = 156)

Parameter	Abbreviation	Unit	Min	Max	Mean	SD	CV
Conductivity	Cond.	μS·cm^-1^	129	875	376	136	0.36
Oxygen content at the bottom	O_2_	%	0.00	53.00	4.20	5.62	1.34
Secchi disk—water transparency	SD	m	0.30	4.10	1.22	0.75	0.61
Total nitrogen	TN	mg N·dm^-3^	0.64	4.86	1.86	0.86	0.46
Total phosphorus	TP	mg P·dm^-3^	0.01	0.92	0.10	0.11	1.00
Multimetric Diatom Index for Lakes	IOJ	-	0.39	0.91	0.67	0.13	0.20
Ecological State Macrophyte Index	ESMI	-	0.05	0.98	0.37	0.18	0.50
Phytoplankton Multimetric for Polish Lakes	PMPL	-	0.07	5.00	2.51	1.32	0.52
Chlorophyll *a*	Chl*a*	μg/l	2.3	175.2	47.7	39.3	0.82

**Table 5:** r-Pearson correlation coefficient between water quality parameters in stratified lakes (*n* = 237; *p < 0.001)

Parameter	SD	Cond.	TN	TP	O_2_
SD	1.000				
Cond.	−0.467*	1.000			
TN	−0.534*	0.620*	1.000		
TP	−0.441*	0.327*	0.435*	1.000	
O_2_	0.390*	−0.121	−0.168	−0.119	1.000

**Table 6:** r-Pearson correlation coefficient between water quality parameters in non-stratified lakes (*n* = 156; **p* < 0.001)

Parameter	SD	Cond.	TN	TP	O_2_
SD	1.000				
Cond.	−0.320*	1.000			
TN	−0.541*	0.588*	1.000		
TP	−0.367*	0.381*	0.442*	1.000	
O_2_	−0.117	−0.084	0.032	−0.019	1.000
